# Cannabidiol Induces Apoptosis and Perturbs Mitochondrial Function in Human and Canine Glioma Cells

**DOI:** 10.3389/fphar.2021.725136

**Published:** 2021-08-11

**Authors:** Chase Gross, Dominique A. Ramirez, Stephanie McGrath, Daniel L. Gustafson

**Affiliations:** ^1^ Department of Clinical Sciences, Colorado State University, Fort Collins, CO, United States; ^2^ University of Colorado Cancer Center, Aurora, CO, United States

**Keywords:** cancer, pharmacology, anti-cancer agents, glioma, brain/CNS tumor, cannabidiol (CBD), mitochondria

## Abstract

Cannabidiol (CBD), the major non-psychoactive compound found in cannabis, is frequently used both as a nutraceutical and therapeutic. Despite anecdotal evidence as an anticancer agent, little is known about the effect CBD has on cancer cells. Given the intractability and poor prognoses of brain cancers in human and veterinary medicine, we sought to characterize the *in vitro* cytotoxicity of CBD on human and canine gliomas. Glioma cells treated with CBD showed a range of cytotoxicity from 4.9 to 8.2 μg/ml; canine cells appeared to be more sensitive than human. Treatment with >5 μg/ml CBD invariably produced large cytosolic vesicles. The mode of cell death was then interrogated using pharmacologic inhibitors. Inhibition of apoptosis was sufficient to rescue CBD-mediated cytotoxicity. Inhibition of RIPK3, a classical necroptosis kinase, also rescued cells from death and prevented the formation of the large cytosolic vesicles. Next, cellular mitochondrial activity in the presence of CBD was assessed and within 2 hours of treatment CBD reduced oxygen consumption in a dose dependent manner with almost complete ablation of activity at 10 μg/ml CBD. Fluorescent imaging with a mitochondrial-specific dye revealed that the large cytosolic vesicles were, in fact, swollen mitochondria. Lastly, calcium channels were pharmacologically inhibited and the effect on cell death was determined. Inhibition of mitochondrial channel VDAC1, but not the TRPV1 channel, rescued cells from CBD-mediated cytotoxicity. These results demonstrate the cytotoxic nature of CBD in human and canine glioma cells and suggest a mechanism of action involving dysregulation of calcium homeostasis and mitochondrial activity.

## Introduction

The prognosis following a glioma diagnosis is notoriously grave, no matter the species. High grade gliomas portend a median survival rate of 14–16 months in human (Stupp et al., 2009) and 2–4 months in canine patients (Rossmeisl et al., 2012). Tumor cell resistance to the currently available multimodal treatments underlies these dismal prognoses. Unfortunately, improvements in patient outcome have not paralleled the medical advances in glioma treatment regimens (Hadziahmetovic et al., 2011; Uhm and Porter, 2017). This highlights the crucial need for further brain cancer treatment research.

Recently, the cannabinoid family, a group of pharmacologically active compounds that primarily interact with specific cannabinoid G-protein coupled receptors (GPCR) (Hermanson and Marnett, 2011), are being rigorously studied for their putative anti-tumorigenic and therapeutically relevant properties. These effects have been observed among a diverse family of cannabinoid compounds, such as naturally occurring cannabinoids, synthetic cannabinoid agonists, and endocannabinoid modulators (Hermanson and Marnett, 2011). In particular, cannabidiol (CBD), a non-psychoactive phytocannabinoid derived from *Cannabis sativa*, has seen accumulating evidence it may function as an antineoplastic agent. CBD has been demonstrated to possess anti-proliferative, cytotoxic, anti-migratory, anti-adhesive, anti-invasive, and anti-neovascularization effects in a wide variety of histogenetically diverse cancer types *in vitro* (Jacobsson et al., 2000; Calvaruso et al., 2012; Massi et al., 2013). The observed cytotoxicity is hypothesized to result from activation of apoptotic and autophagic pathways, with a particular interest in the involvement of the Voltage Dependent Anion Channel 1 (VDAC1) ion channel (Calvaruso et al., 2012; Rimmerman et al., 2013). However, the exact mechanism(s) of these antineoplastic effects remain nebulous.

Current use of cannabinoids in cancer therapy is predominantly through non-prescription cannabis, including tetrahydrocannabinol (THC) or related cannabinoid agonists for alleviation of unwanted concomitant effects of chemo- and radiotherapeutic regimens. While THC and similar cannabinoids are effective in palliative uses, patients often experience unwanted psychoactive effects, tolerance to, and potentially dependence upon these treatments. CBD, in addition to being non-psychotropic, exhibits several therapeutically beneficial effects such as analgesia, anxiolysis, and anti-inflammatory properties (Dzierżanowski, 2019).

Cannabinoids exert their pharmacological effects through a wide variety of receptor-dependent and independent interactions. These interactions are capable of modifying signaling pathways that are crucial in tumorigenesis, cancer growth, and metastasis (Kendall and Yudowski, 2016). The cannabinoid-specific GPCRs CB1 and CB2 make up the predominant population of the endocannabinoid system (Zou and Kumar, 2018). CB1 is one the most common GPCR in the human and canine brain (Zou and Kumar, 2018; Silver, 2019). CBD has low binding affinity to the cannabinoid receptors, but is capable of modifying them and exerting effects through a diverse cornucopia of other receptors including, but not limited to: TRPV1, mTOR, PPAR, GABA, and 5HT receptors (Zador and Wollemann, 2015; O'Sullivan, 2016; Bakas et al., 2017; De Gregorio et al., 2019). The TRPV1 receptor has been implicated in many of CBD’s clinical effects such as analgesia (Zador and Wollemann, 2015). The variable and ubiquitous nature of the cannabinoid signaling system in the brain results in diverse modification of crucial cellular pathways and suggests a potential for utilizing CBD as a targetable pharmacophore. Further detailing of these interactions will provide invaluable information about CBD’s effects on cells and reveal further potential of CBD as a therapeutic.

Several studies have demonstrated the remarkable similarities between canine and human gliomas, including MRI characteristics, histological and immunohistochemical features, genetic conservation, and immunological responses (Thomas et al., 2009; Herranz et al., 2016; Koehler et al., 2018). Thus, spontaneously occurring canine gliomas serve as an ideal surrogate for human brain tumors. The present study explores the anti-cancer phenomena of CBD in human and canine glioma cells *in vitro* and details the biochemical mechanisms responsible as a foundation for development of CBD as a potential clinical therapeutic.

## Materials and Methods

### Cell Culture and Reagents

Two canine glioma cells lines (J3TBG, SDT3G) were provided by UC Davis. J3TBG was derived from a grade III astrocytoma and SDT3G from a grade III glioblastoma sample (York et al., 2018). The human cell line U87MG was purchased from the University of Colorado Anschutz Tissue Culture Shared Resource (PMTSR). The human cell line U373MG Uppsala was purchased from the ECACC. All cell lines were cultured in Minimum Essential Media Eagle (MEM; Corning, Inc. Corning, NY) supplemented with 10% FBS, 100 U/ml streptomycin, 4.5 g/L glucose and 10 mM sodium pyruvate at 37°C, in 5% CO_2_. Cell cultures were maintained in semi-confluent monolayers and passaged 1–2 times per week. Cell lines were maintained for no more than 30 passages, at which time a new stock was utilized. All cell lines were verified by STR (Short Tandem Repeats) at the Flint Animal Cancer Center Cell Line Validation Core at Colorado State University. Cells were verified to be mycoplasma-free *via* PCR prior to transduction with the IncuCyte™ NucLight Red lentiviral system (Essen Bioscience Inc., Ann Arbor, MI) and were selected with puromycin following transduction. CBD was provided by Extract Labs (Boulder, CO) and analyzed for purity. Two forms of CBD-dominant products were provided: highly-purified CBD isolate (>99.9% pure cannabidiol) and CBD extract (96% CBD, 4% cannabinoid diverse). Both forms of CBD were prepared as a stock solution in DMSO, and later diluted to 100x working stocks in 50/50 ethanol/ultra-pure water. z-VAD-fmk (final concentration of 50 µM (Lincoln et al., 2018), dissolved in 50/50 ethanol/ultra-pure water), necrostatin-1 (final concentration of 100 µM (Huang et al., 2013), dissolved in DMSO/ultra-pure water), and VX-765 (final concentration of 50 µM (McKenzie et al., 2018), dissolved in 50/50 ethanol/ultra-pure water) were purchased from Sigma-Aldrich (MilliporeSigma; Burlington, MA). GSK’872 (final concentration of 1 µM (Mandal et al., 2014), dissolved in 50/50 ethanol/ultra-pure water), 4,4’-Diisothiocyano-2,2′-stilbenedisulfonic acid (DIDS) (final concentration of 50 µM (Benitez-Rangel et al., 2015), dissolved in 50/50 ethanol/ultra-pure water), and 5-IRTX (final concentration of 1 µM) (Stampanoni Bassi et al., 2019), dissolved in 50/50 ethanol/ultra-pure water) were purchased from Tocris Bioscience (Bristol, United Kingdom). Concentrations of reagents were determined using previously published inhibitory values.

### Cytotoxicity Assays

Cells were seeded in 96-well Nunclon Delta Surface Plate (Thermo-Scientific, Rockford, IL) at densities to ensure mid-late log phase growth at 96 h (3,000 cells/well). Cells were allowed to adhere for a minimum of 4 h before dosing. Cell viability was quantified utilizing the membrane impermeable dye YOYO-1 (Thermo-Scientific, Rockford, IL) at 100 nM. Assays were imaged every 3 h using IncuCyte™ Zoom (Essen Bioscience, Ann Arbor, MI). At 96 h, the percentage of dead cells was calculated by dividing the measured final green fluorescence (a surrogate of cell death) by the sum of the final red fluorescence per mm^2^ (a surrogate of cell viability) and green fluorescence per mm^2^. Subgroups in biological replicates were performed in technical triplicate. Bars represent the mean across *n* = 4 biological replicates, and the error bars represent the standard deviation between biological replicates.

### Seahorse XFe24 Assays

Cells were resuspended in Seahorse XF DMEM (Agilent Technologies, Santa Clara, CA) supplemented with 100 μg/ml streptomycin, 4.5 g/L glucose, and 10 mM sodium pyruvate. Cells were plated at an empirically determined density of 7.5 × 10^4^ cells/well in Cell-Tak (Corning, Inc. Corning, NY) treated XF24 cell culture microplates (V7-PS; Seahorse Bioscience, North Billerica, MA). Cells were then spun at 500*g for 5 min and allowed to equilibrate in a 37°C non-CO_2_ incubator immediately before metabolic flux analysis. Oxygen Consumption Rate/Extracellular Acidification Rate (OCR/ECAR) were then measured by the Agilent Seahorse XFe24 Analyzer (Agilent Technologies, Santa Clara, CA) under basal conditions for 30 min and post-injection of compounds. The data were then validated by injection of oligomycin (18.5 µM), rotenone/antimycin A (6.6 µM), and carbonyl cyanide *m*-chlorophenyl hydrazine (6 µM). Biological replicates were performed in technical triplicate. Data shown are from *n* = 3 independent biological replicates until x = 267 min, the subsequent data is from *n* = 2 independent biological replicates.

### Live Cell Imaging and Mitochondria Staining

NucLight-Red transfected cells (SDT3G and U373MG) were plated onto Cellvis 8-well chambered cover glass (C8-1.5H-N, Cellvis, Mountain View CA) in DMEM at a density to ensure 50–70% confluency at the time of dosing for live cell imaging studies. Cells were dosed with CBD after adhering to the cover glass. Incubation times as indicated were staggered such that all incubations would end at the time of imaging. Cells were stained with both MitoTracker Deep Red FM (M22426, ThermoFisher, Waltham MA) and Hoescht (33342, ThermoFisher, Waltham MA) prior to imaging. During the staining and imaging, cells were maintained in phenol red-free supplemented DMEM, termed “imaging media,” containing the following: penicillin/streptomycin (15140163, Gibco), l-glutamine (A2916801, Gibco), essential (11130–051, Gibco) and nonessential (11140–076, Gibco) amino acids all diluted to a final of 1x from the stock, and sodium bicarbonate (S233-500, Fisher, Waltham MA) at a final concentration of 1.5%. Before the end of the incubation, cells were washed once with imaging media and were stained with 50 nM MitoTracker diluted in imaging media for 15 min at 37°C followed by 10 μM Hoescht for 5 min at room temperature. CBD was maintained at appropriate concentrations for the staining and imaging steps. Live cell images were then visualized using an Olympus IX83 (Olympus, Tokyo, Japan) confocal microscope and Hamamatsu ORCA-R2 (Hamamatsu Photonics, Shizuoka, Japan) digital camera. Visual information corresponding to nuclear RFP (NucLight-Red) were captured for analysis but were excluded from the publication images.

### ATP Luminescence Assay

Cellular ATP levels were measured using a luminescent ATP detection assay kit according to the manufacturer’s instruction (ab113849, Abcam, Cambridge, MA). Cells were seeded at a density of 3,000 cells/well, allowed to adhere for 4 h, and dosed in the same method as the cytotoxicity assays. Luminescence of luciferase was read *via* a BioTek Synergy HTX 96-well microplate reader (Biotek, Winooski, VT). ATP concentration for individual wells was interpolated *via* standard curve in GraphPad Prism (GraphPad Software, Santa Clara, CA). Luminescence of cell culture media was subtracted from each well. Data was then averaged, corrected for proliferation, and subsequently normalized to their respective controls. Biological replicates were performed in technical triplicate. Data shown are from *n* = 2 independent biological replicates.

### Resazurin Metabolic Assay

Resazurin sodium salt was purchased from Sigma-Aldrich (MilliporeSigma; Burlington, MA). Cells were seeded at a density of 3,000 cells/well, allowed to adhere for 4 h, and were subsequently dosed with varying conditions (0–20 µg) for a final well volume of 180 µL. Wells were dosed with same methods as cytotoxicity assays. After 96 h, 20 µL of resazurin reagent was added to each well for a final well volume of 200 µL. Cells were then incubated for 4 h in standard culture conditions. Resazurin reagent reduction, a surrogate of cell metabolism, was measured *via* fluorescence (excitation: 540 nm; emission: 590 nm) in a 96-well microplate reader. DMEM control fluorescence was subtracted for each well. Fluorescence data was averaged and subsequently normalized to their respective controls. Assays were performed in biological and technical triplicate: the bars represent the mean across biological replicates and the error bars represent the Standard Deviation between biological replicates.

### Data Analysis

Statements of technical and biological replicates for each assay are included in the respective method subsection and in the figure legends. Statistical tests were only performed for assays with biological replicates of n ≥ 3. All tests used are named in the figure legend with the associated data. Data for statistical analyses were not explicitly tested for normality, nor were variances between compared groups tested to be statistically significantly different. All measures of variation are reported as standard deviation (SD). Statistical analyses were carried out using GraphPad Prism 8 (GraphPad Software, La Jolla, CA, United States). An * indicates a calculated *p*-value of ≤0.05.

## Results

### CBD Is Anti-proliferative, Cytotoxic, and Induces the Formation of Intracellular Vesicles in Glioma Cells

**FIGURE 1:**
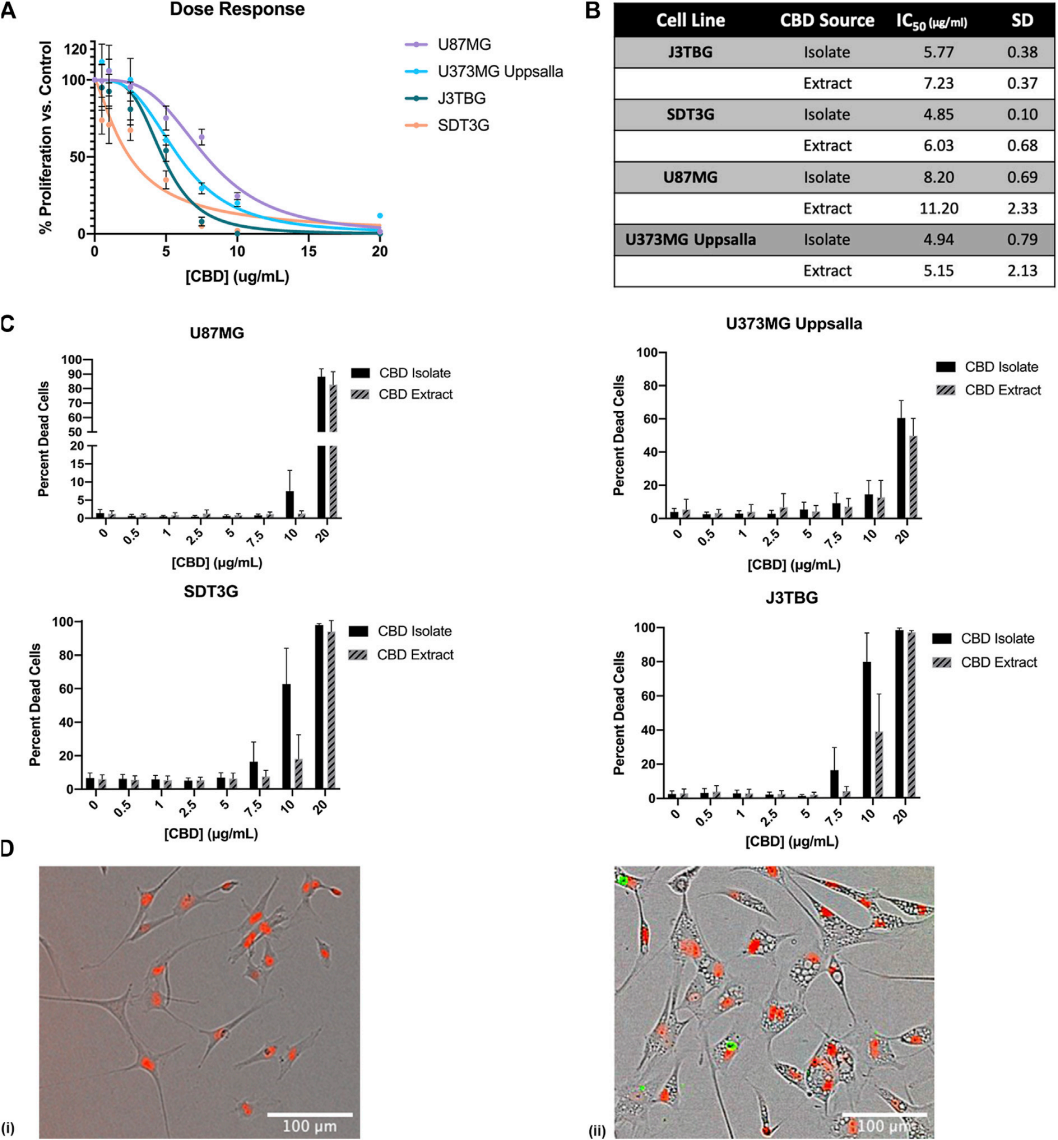
CBD is antiproliferative and cytotoxic to glioma cell lines. **(A)** U87MG, U373MG, J3TBG, and SDT3G Glioma cells were treated with CBD isolate (0–20 μg/ml) for 96 h. Cell proliferation and dose response were measured *via* IncuCyte™ Red Object Count. Data are from *n* = 4 independent repeated experiments. Individual points expressed as mean ± SD, curve fit through a non-linear, variable slope regression **(B)** IC_50_ values were determined from the curve fits *via* GraphPad Prism for both CBD isolate and extract at 96 h. Data are from *n* = 4 independently repeated experiments **(C)** U87MG, U373, J3TBG, and SDT3G Glioma cells were treated with CBD isolate or extract (0–20 μg/ml) for 96 h. Cytotoxicity was determined using the green fluorescent dye, YOYO-1. Data are from *n* = 4 independently repeated experiments**,** expressed as mean ± SD **(D)** SDT3G glioma cells treated with 0 μg/ml CBD after 48 h (i), SDT3G Glioma cells featuring formation of intracellular vesicles after 48 h of treatment with 7.5 μg/ml CBD isolate (ii) At 48 h, cell death is not occurring at this concentration. Red fluorescence represents nuclei of viable cells. Images were captured through a 10x Nikon objective.

To investigate the effect of CBD treatment on cell line proliferation and cytotoxicity, we monitored cell viability *via* the IncuCyte™ in a selection of glioma cells including canine cell lines SDT3G and J3TBG and human cell lines U87MG and U373MG Uppsala (Figure 1). Two sources of CBD-dominant compounds were used, >99.9% pure CBD isolate, and cannabinoid diverse CBD extract (96% CBD). Cell proliferation decreased starkly following CBD isolate treatment (Figures 1A,B). The cytotoxicity of CBD against human and canine gliomas was also confirmed using the live/dead dye YOYO-1 (Figure 1C). This measure of cytotoxicity confirmed cell death at the three highest concentrations (7.5, 10, 20 μg/ml) for both sources of CBD. In all cases, glioma cells appeared to be marginally more sensitive to CBD isolate than CBD extract (Figures 1B,C); later experiments were conducted solely with the highly-purified CBD isolate. Treatment with cytotoxic concentrations of CBD invariably induced the formation of intracellular vesicles (Figure 1D) across all cell lines.

### CBD Induces Apoptosis With Involvement From the Necroptotic Kinase RIPK3

**FIGURE 2:**
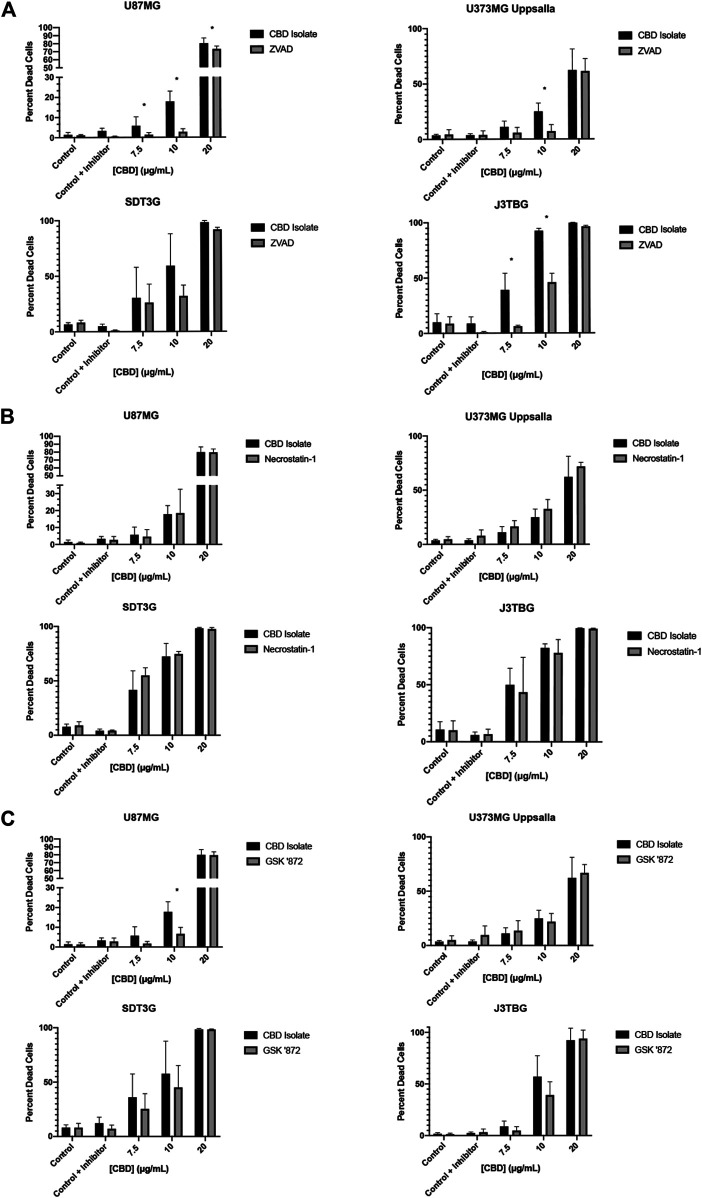
CBD induces apoptosis with contribution from the protein kinase RIPK3. **(A)** U87MG, U373MG, J3TBG, and SDT3G glioma cells were treated with CBD isolate (0–20 μg/ml) and zVAD (50 µM) concurrently for 96 h and cytotoxicity determined using the green fluorescent dye, YOYO-1. Representative of *n* = 3 independent experiments expressed as mean ± SD. *P (two-tailed *t*-test). U87MG: 7.5 (0.03), 10 (<0.001), 20 (0.03). U373MG Upsalla: 10 (<0.001). J3TBG: 7.5 (<0.001), 10 (<0.001), 20 (<0.001) **(B)** Cytotoxicity of glioma cells treated with CBD isolate (0–20 μg/ml) and necrostatin-1 (100 µM) concurrently for 96 h. Representative of *n* = 3 independent experiments expressed as mean ± SD. **(C)** Cytotoxicity of glioma cells treated with CBD isolate (0–20 μg/ml) and GSK ‘872 (1 µM) concurrently for 96 h. Data shown are from *n* = 3 independently repeated experiments expressed as mean ± SD. *P (two-tailed *t*-test). U87MG: 10 (<0.001).

Next, we investigated the mode of cell death involved in CBD-mediated cytotoxicity. We investigated three primary means of cell death: apoptosis, necroptosis, and pyroptosis. To observe the contribution of apoptosis, glioma cell lines were treated with CBD (0–20 μg/ml) and zVAD-fmk (zVAD) (50 µM), a pan-caspase inhibitor for 96 h (Figure 2A). At cytotoxic CBD concentrations (7.5, 10, 20 μg/ml), zVAD prevented cell death and rescued cell viability across all cell lines. To observe the contribution of necroptosis, glioma cell lines were treated with CBD and necrostatin-1 (100 µM), a RIPK1 inhibitor. At cytotoxic CBD concentrations, necrostatin-1 was unable to rescue CBD-induced cell death (Figure 2B). As part of our necroptosis investigation, we also inhibited RIPK3, a classically associated necroptotic kinase. Glioma cells were treated with CBD and GSK872 (1 µM), a RIPK3 inhibitor and putative inhibitor of necroptosis. At cytotoxic CBD concentrations, GSK872 rescued cells from CBD-induced cell death, but not to the same degree as zVAD, (Figure 2C), and prevented the formation of CBD-mediated intracellular vesicles (Supplementary Figure S1A). Finally, we investigated the contribution of caspase-1 mediated pyroptosis as a mode of CBD-mediated cytotoxicity. U87MG and J3TBG Glioma cells were treated with CBD and VX-765 (Belnacasan, 50 µM), a caspase-1 inhibitor. VX-765 was unable to rescue CBD-induced cell death in either cell line (Supplementary Figure S1B).

To characterize the timeline of CBD-mediated cytotoxicity, glioma cell lines were treated with CBD (0–20 μg/ml) for 96 h. In an effort to rescue cells from CBD-mediated cytotoxicity, sub-groups had the CBD-supplemented media replaced with fresh CBD-free media at 24 and 48 h. Across all cell lines, media replacement at 24 h was able to generally rescue cell viability, while the media replacement at 48 h appeared similar to the no replacement control. Our data suggest that a commitment to cell death occurs between 24- and 48-h post CBD exposure (Supplementary Figure S1C). Observed mild discrepancies in rescue between concentrations/cell lines are likely due to the methods used during the wash-off experiments. Cells were likely accidently and unavoidably aspirated, or disturbed during replacement with CBD-free medium. We suspect this discrepancy does not reflect the true biologic phenomena.

### CBD Impairs Mitochondrial Activity at Sub-cytotoxic Concentrations

**FIGURE 3:**
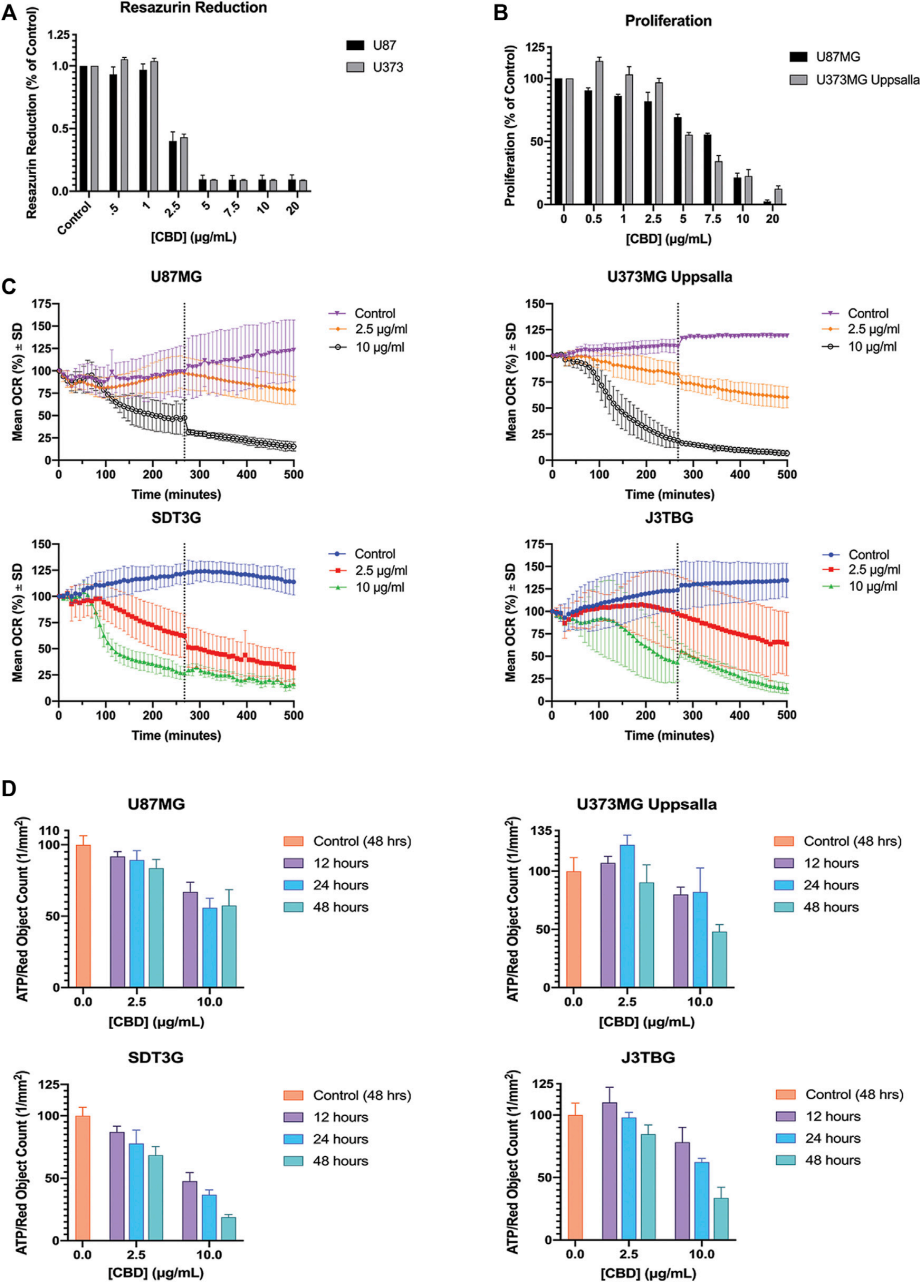
CBD perturbs mitochondrial activity at lethal and non-lethal concentrations. **(A)** Glioma cell lines U87MG and U373MG Uppsala were treated with CBD isolate (0–20 ug/mL) for 96 h. Cell viability was determined *via* resazurin-based metabolic assay. Data (arbitrary fluorescence units) were normalized to the vehicle treated control. Representative of *n* = 3 independent experiments, expressed as mean ± SD. **(B)** Glioma cell lines U87MG and U373MG Uppsala were treated with CBD isolate (0–20 μg/ml) for 96 h. Cell proliferation was measured *via* IncuCyte™ Red Object Count. Data shown are from *n* = 3 independently repeated experiments, expressed as mean ± SD. **(C)** Glioma cells were measured for baseline oxygen consumption for 30 min, then treated with CBD at lethal (10 μg/ml) and non-lethal (2.5 μg/ml) concentrations. OCR was monitored for 500 min using the SeaHorse XF24. Data shown are from *n* = 3 independent biological replicates until x = 267 min (vertical dashed grey line) at which point the subsequent data are from *n* = 2 independent biological replicates. Data are expressed as mean ± SD. **(D)** U87MG, U373, J3Tbg, and SDT3G Glioma cells were treated with CBD isolate (2.5, 10 μg/ml) for 12, 24, and 48 h. Cellular ATP production was determined *via* ATP luminescence kit. Data (fluorescence) were interpolated *via* standard curve, corrected for proliferation (Red Object Count *via* Incucyte™) and normalized to the 48-h vehicle treated control. Data shown are from *n* = 2 independent experiments. Data are expressed as mean ± SD.

U87MG and U373MG Uppsala human glioma cells were treated with CBD for 96 h and cytotoxicity was measured using two methods: (Figure 3A): resazurin reduction (*surrogate of cell viability measuring mitochondrial function*); and (Figure 3B) cell proliferation using red fluorescence on the IncuCyte™. Treatment with CBD drastically impairs mitochondrial-based resazurin reduction, suggesting sensitivity at much lower concentrations of CBD than that determined by IncuCyte™. Thus, CBD impairs mitochondrial activity at sub-cytotoxic concentrations without altering cell proliferation or death. This finding was confirmed *via* Seahorse XFe24 analysis of mitochondrial OCR (Figure 3C) and ECAR (Supplementary Figure S1D) post-CBD treatment. In all glioma cell lines, we observed a decrease in oxygen consumption reflecting inhibition of mitochondrial respiration that occurs long before detectable cytotoxicity. Treatment with CBD altered cellular ATP production at both sub-cytotoxic and lethal concentrations in a dose-dependent and time-sensitive manner. In all glioma cell lines, a decrease in ATP production after treatment with lethal and sub-cytotoxic concentrations of CBD was observed, further suggesting compromise of mitochondrial function (Figure 3D).

### Treatment With a Lethal CBD Concentration Produces Ultrastructural Changes in the Mitochondria

**FIGURE 4:**
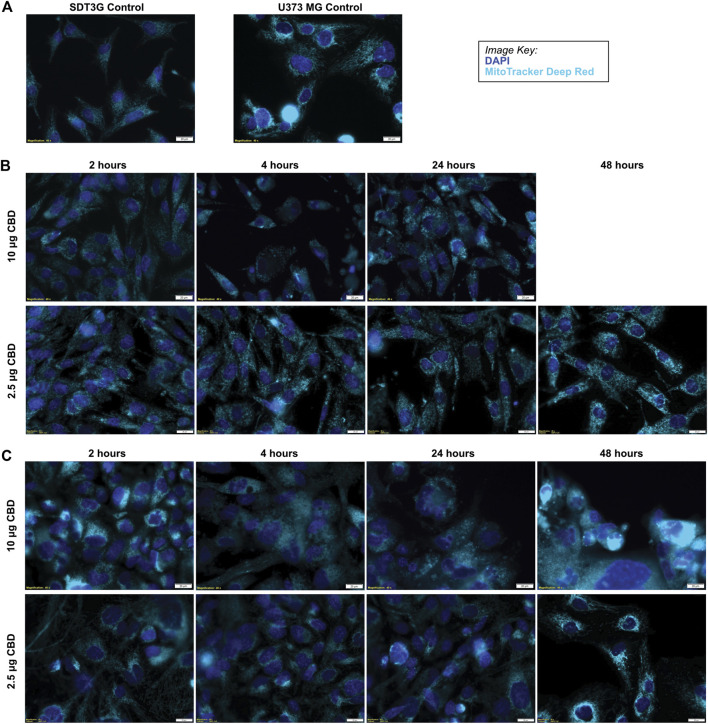
CBD causes morphological changes in mitochondria. **(A)** Untreated SDT3G and U373 MG cells demonstrate a fibrillar mitochondrial network. Following treatment with CBD, both SDT3G **(B)** and U373 MG **(C)** cells show dramatic changes in mitochondrial morphology within 2 hours of 10 μg/ml CBD treatment that is not observed at 2.5 μg/ml CBD. Nuclei are represented by dark blue and mitochondria are represented by cyan pseudo colors.

SDT3G and U373MG cells were stained with MitoTracker Deep Red FM and visualized by live cell imaging to investigate morphological changes related to CBD treatment. The untreated cell lines displayed a fibrillar mitochondrial network distributed throughout the cell (Figure 4A). Each cell line was then treated with a cytotoxic (10 μg/ml) and non-cytotoxic (2.5 μg/ml) dose of CBD and visualized at time points throughout the known cell-death decision time frame (Supplementary Figure S1C.). Within 2 h post-treatment, both SDT3G (Figure 4B) and U373MG (Figure 4C) cells showed dramatic alteration of mitochondrial morphology and disruption of the fibrillar network but only at the high CBD concentration. At this concentration the mitochondria were very large and circular. Images for SDT3G at 48 h post-high dose CBD treatment could not be captured because no viable cells remained. This phenomenon was not observed at the low CBD concentration where the baseline mitochondrial morphology and network were largely preserved throughout the 48-h time frame. The spherical mitochondria seemingly correspond to the large vesicles observed by brightfield in the IncuCyte™ (Figure 1D [ii]). J3TBG and U87MG cell lines were not included in this experiment series due to the morphology of the cells; the large nuclei and scant cytoplasm prevented adequate visualization of the mitochondrial morphology.

### CBD-Mediated Cytotoxicity Involves VDAC1 but Not TRPV1

**FIGURE 5:**
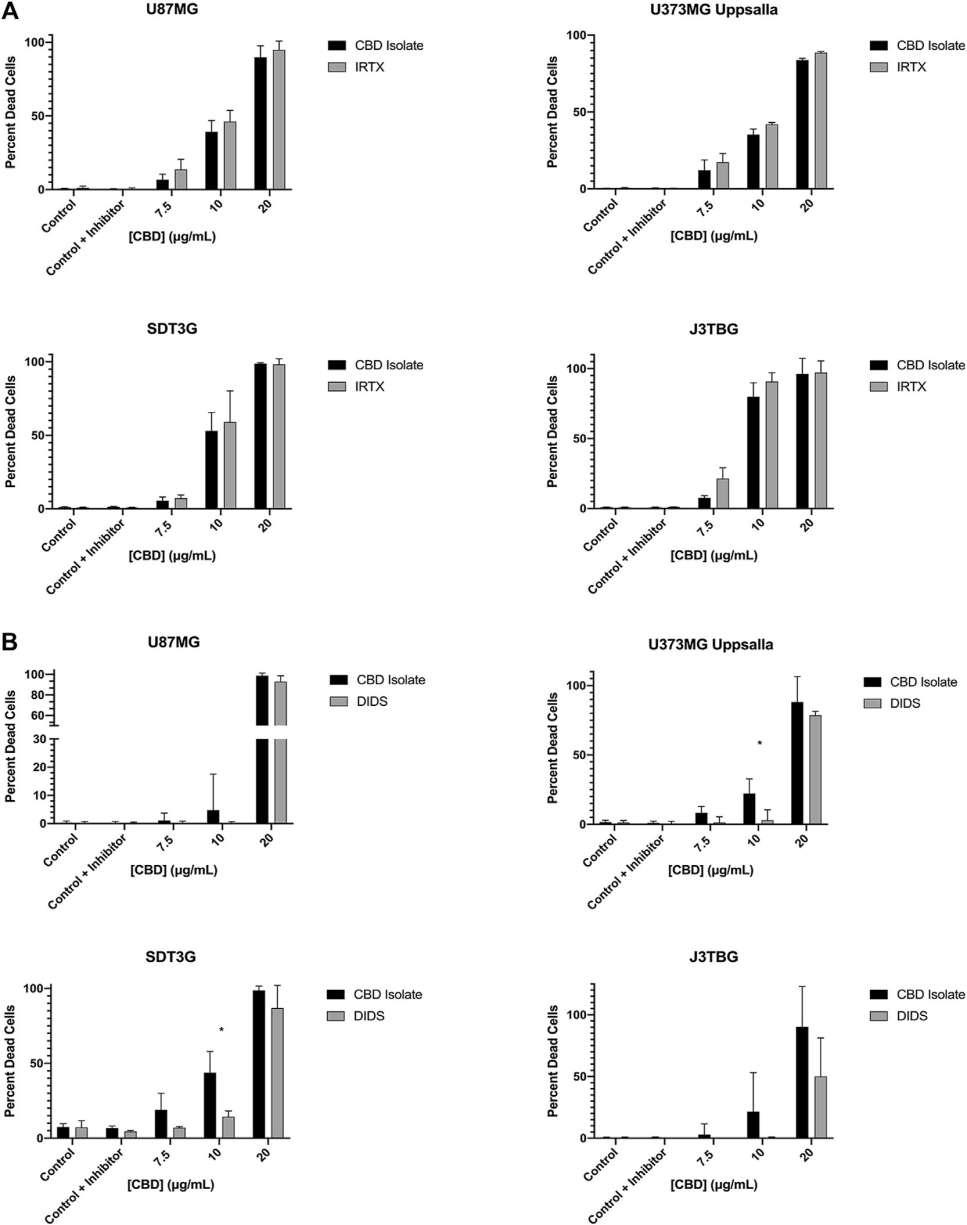
CBD mediated cytotoxicity involves VDAC1 but not TRPV1. **(A)** U87MG, U373MG, J3TBG, and SDT3G Glioma cells were treated with CBD isolate (0–20 μg/ml) and IRTX (1 µM) concurrently for 96 h and cytotoxicity determined using the green fluorescent dye, YOYO-1. Representative of *n* = 3 independent experiments expressed as mean ± SD. *P (two-tailed *t*-test). **(B)** Cytotoxicity of glioma cells treated with CBD isolate (0–20 μg/ml) and DIDS (50 µM) concurrently for 96 h. Data shown are from *n* = 3 independent experiments expressed as mean ± SD. *P (two-tailed *t*-test). U373MG Upsalla: 10 (0.02). SDT3G: 10 (0.003).

Cell Lines U87MG, U373MG Uppsala, SDT3G, and J3TBG were treated concurrently with CBD (0–20 μg/ml) and IRTX (1 µM), a known inhibitor of TRPV1 (Figure 5A). At cytotoxic CBD concentrations, inhibition of TRPV1 was unable to rescue cell viability. Cell lines were treated with CBD (0–20 μg/ml) and DIDS (50 µM), a known inhibitor of the VDAC1 (Figure 5B). At cytotoxic CBD concentrations, inhibition of VDAC1 rescued cell viability similar to that observed following inhibition of apoptosis.

## Discussion

Recent studies with cannabinoids have begun to identify potential activities that may be exploited for anticancer therapy. A wide range of antineoplastic effects on an array of histogenetically diverse cancers, relatively safe therapeutic index, and growing popularity in medicine and society makes them ideal candidates for further research. CBD has consistently induced apoptotic cell death *in vitro* and *in vivo* for many types of neoplasia (Shrivastava et al., 2011; Wu et al., 2018; Jeong et al., 2019). While the exact mechanism of action underlying this cytotoxicity has not been fully elucidated, it has been hypothesized that mitochondrial dysfunction precipitated by altering VDAC1 channel activity is central to the observed antiproliferative, apoptotic, and other antineoplastic signaling pathways (Rimmerman et al., 2013; Magrì et al., 2018; Olivas-Aguirre et al., 2019).

In this present study, we demonstrate that highly purified CBD isolate reduced proliferation and induced caspase-mediated cell death, suggestive of apoptosis, in both canine glioma cell lines SDT3G and J3TBG as well as the human glioma cell lines U87MG and U373MG Uppsala. The commitment to CBD-induced apoptotic cell death involves a critical cell fate decision between 24 and 48 h. Our data also demonstrate that RIPK3, a protein kinase classically associated with necroptosis (Orozco and Oberst, 2017), plays an impactful role in CBD-mediated cytotoxicity and formation of intracellular vesicles, suggesting a non-canonical function of the kinase. RIPK3 has been implicated as a proapoptotic adaptor after the formation of the necrosome, particularly when the core necroptotic machinery component Mixed Lineage Kinase domain-like protein (MLKL) is absent (Mandal et al., 2014; Newton et al., 2014; Orozco and Oberst, 2017). Interestingly, the domestic dog (*Canis lupus familiaris*) does not possess the MLKL gene (Dondelinger et al., 2016), perhaps highlighting an even more central role for RIPK3 in determining CBD sensitivity in canine gliomas. In addition, RIPK3 has also been implicated in the execution of mitochondrial-mediated apoptosis in cardiac myocytes during hypoxic insult *via* inhibition of the FUNDC1 pathway, and thereby inhibition of mitophagy, a selective degradation of damaged mitochondria (Zhou et al., 2017). Future investigation of these potential phenomena may help to clarify the exact biophysical mechanisms behind CBD-mediated cytotoxicity in glioma cells.

The effects of the cannabinoid family are extremely diverse and their exact mechanisms tend to be incompletely characterized, particularly in the oncology arena. CBD’s interaction with the TRP receptor family has been an exciting line of research and has been implicated in many of CBD’s observed therapeutic effects (Costa et al., 2004; Zador and Wollemann, 2015; Whalley et al., 2018). The TRP family is a multigene, superfamily of ion channels that produce a range of physiological effects (Samanta et al., 2018). In particular, the TRPV1 receptor, a calcium ion channel found to be localized to the IMM of the mitochondria in nonneuronal cells (Kim et al., 2017), appears to have diverse cellular effects after exposure to CBD, with variability dependent on cell type, physiologic state, and CBD dosage (Costa et al., 2004; Zador and Wollemann, 2015; Whalley et al., 2018). TRPV1 activation induces cell death after treatment with the TRPV1 agonist, capsaicin, *via* a rapid increase in intracellular calcium (Ramírez-Barrantes et al., 2018). CBD-induced intramitochondrial calcium overload has been implicated in the formation of the fatal mitochondrial permeability transition pore (mPTP) and subsequent CBD-mediated cell death, independent of the CB1/CB2 receptors (Rimmerman et al., 2013; Wu et al., 2018; Olivas-Aguirre et al., 2019). Here, we sought to investigate if TRPV1 channel-mediated increases in mitochondrial calcium flux after CBD treatment might explain our observations. Pharmacologic inhibition of the TRPV1 receptor with IRTX, however, did not produce any protective effects across cell lines, suggesting the TRPV1 receptor is not involved with CBD-mediated cell death in our model. The VDAC1-Mitochondrial Calcium Uniporter complex, an outer and inner mitochondrial membrane mediator of calcium uptake from the cytosol, is being investigated in CBD mediated cell death (Rimmerman et al., 2013). Research has demonstrated colocalization and direct interaction of CBD and VDAC1, heavily implicating this channel in observed cellular energy pathologies (Rimmerman et al., 2013). Pharmacologic inhibition of VDAC1 with DIDS produced stark protective effects on cell viability, suggesting that VDAC1 is a major contributor to CBD-mediated cell death. While pharmacologic inhibition of VDAC1 produced results in support of our hypothesis, we cannot exclude other off-target effects of DIDS, such as caspase inhibition (Benitez-Rangel et al., 2015). Ideally, future studies will investigate individual genetic knockout or knockdown of the TRPV1, MCU, and VDAC1 channels to clarify their role in CBD-mediated pathogenesis.

Targeted dysregulation of mitochondrial bioenergetics has become a leading hypothesis for the observed CBD anticancer effects (Rimmerman et al., 2013; Wu et al., 2018; Jeong et al., 2019; Olivas-Aguirre et al., 2019). In this present study we show a significant perturbation of mitochondrial OCR and ECAR soon after treatment with both lethal and non-lethal doses of CBD that appears to persist for almost 6 h post-treatment. Previous studies have demonstrated CBD’s ability to alter mitochondrial membrane potential, (Rimmerman et al., 2013; Olivas-Aguirre et al., 2019), which is delicately tied to Complex I function. These findings are corroborated qualitatively by the dramatic ultrastructural changes in mitochondria seen as soon as 2 h post-treatment with a lethal dose of CBD (10 μg/ml). Across all cell lines, these observed ultrastructural changes at 2-h post-lethal treatment support the significant drop of OCR observed *via* the Seahorse XFe24 at approximately 150 min (120 min post treatment). We hypothesize, at lethal concentrations, CBD alters permeability of the VDAC1 channel to facilitate a rapid influx of calcium into the mitochondria which leads to a loss of mitochondrial function due to perturbation of membrane potential, release of reactive oxygen species (ROS), and depletion of ATP. This dysfunction also results in the formation of the mPTP, mitochondrial swelling, and subsequent cell death. Previous studies have demonstrated a cellular-protective effect of cyclosporine A, a mPTP inhibitor (Sharov et al., 2007) but not with FK-506, a calcineurin inhibitor (Dumont, 2000). The respective mechanism of action of these two drugs suggests the involvement of the mPTP in CBD-mediated cell death (Wu et al., 2018).

In previous CBD studies, utilization of the MTT, resazurin, or other related metabolic assays has been the standard for assessing cell viability and cytotoxicity analysis (Shrivastava et al., 2011; De Petrocellis et al., 2013; Solinas et al., 2013; Lukhele and Motadi, 2016). The stark disparity between our resazurin reduction fluorescence data and cell proliferation data suggest that mitochondrial Complex I function is compromised at doses of CBD that have little to no effect on cell viability. Therefore, cell viability assays dependent on mitochondrial respiration may over-estimate CBD-mediated cytotoxicity and our results discourage continued use of these assays for CBD related studies. This also raises the tantalizing possibility that CBD might have therapeutic effects at sub-cytotoxic doses that could prove synergistic with other standard interventions such as temozolomide, external beam radiation, and perhaps ascorbic acid.

The grave prognosis and relatively ineffective currently available treatment strategies make further glioma therapeutic research crucial. The growing body of knowledge of the pharmacology, anticancer effects, and other therapeutically relevant properties of cannabidiol reveal the exciting potential of CBD as a potential clinical therapeutic.

## Data Availability

The original contributions presented in the study are included in the article/Supplementary Material, further inquiries can be directed to the corresponding author.
