# Supplementary material for: Cannabidiol Induces Apoptosis and Perturbs Mitochondrial Function in Human and Canine Glioma Cells
Source: Front Pharmacol. 2021 Aug 11;12:725136. doi: 10.3389/fphar.2021.725136 (PMC8385407; doi:10.3389/fphar.2021.725136)
Supplement: Supplementary file 1 [file Image1.pdf]

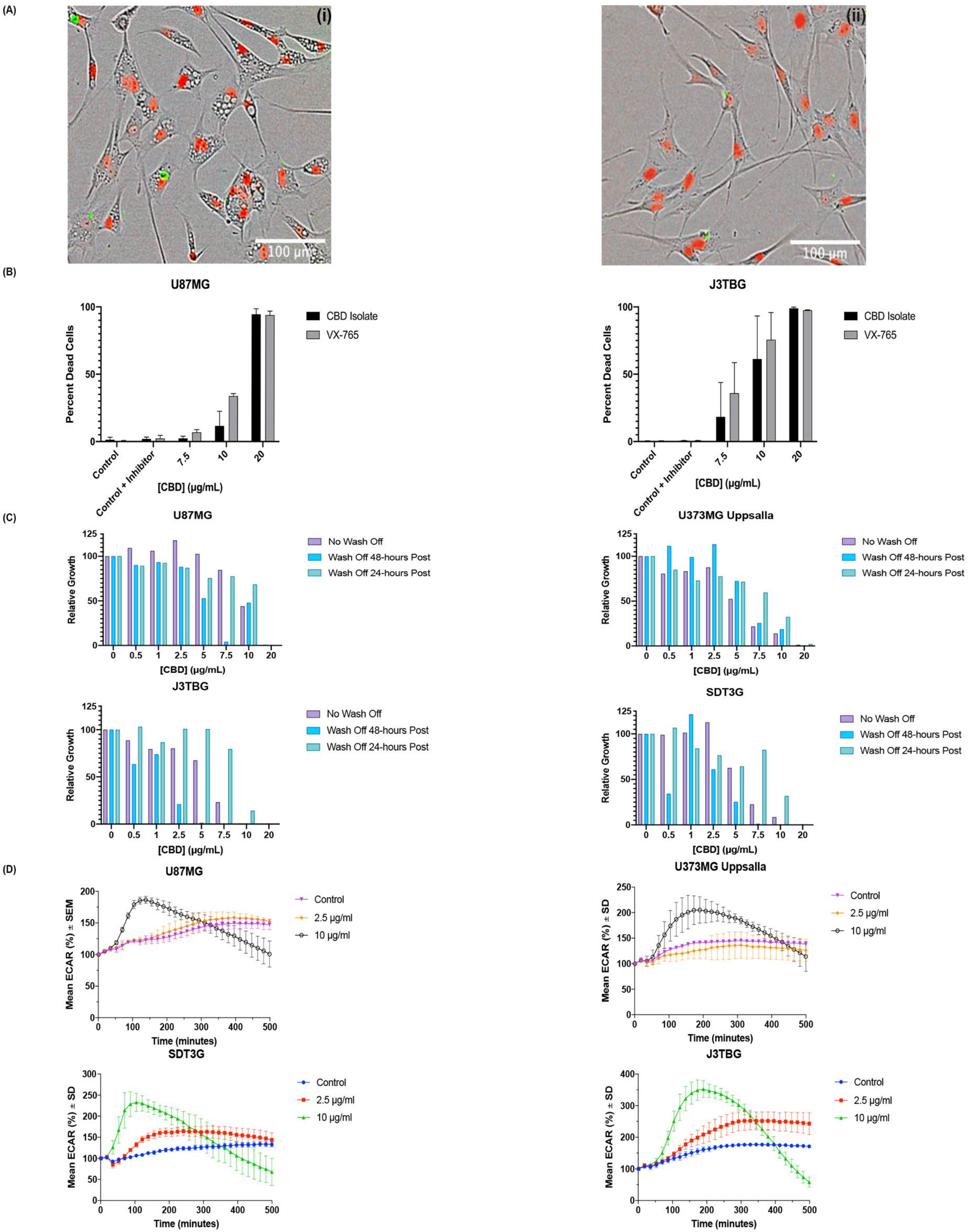
(A)
(i)
100 μm
(ii)
100 μm
(B)
U87MG
Percent Dead Cells
CBD Isolate
VX-765
Control
Control + Inhibitor
7.5
10
20
[CBD] (μg/mL)
J3TBG
Percent Dead Cells
CBD Isolate
VX-765
[CBD] (μg/mL)
(C)
U87MG
Relative Growth
No Wash Off
Wash Off 48-hours Post
Wash Off 24-hours Post
[CBD] (μg/mL)
U373MG Uppsalla
Relative Growth
No Wash Off
Wash Off 48-hours Post
Wash Off 24-hours Post
[CBD] (μg/mL)
J3TBG
Relative Growth
No Wash Off
Wash Off 48-hours Post
Wash Off 24-hours Post
[CBD] (μg/mL)
SDT3G
Relative Growth
No Wash Off
Wash Off 48-hours Post
Wash Off 24-hours Post
[CBD] (μg/mL)
(D)
U87MG
Mean ECAR (%) ± SEM
Control
2.5 μg/ml
10 μg/ml
Time (minutes)
U373MG Uppsalla
Mean ECAR (%) ± SD
Control
2.5 μg/ml
10 μg/ml
Time (minutes)
SDT3G
Mean ECAR (%) ± SD
Control
2.5 μg/ml
10 μg/ml
Time (minutes)
J3TBG
Mean ECAR (%) ± SD
Control
2.5 μg/ml
10 μg/ml
Time (minutes)

Supplementary Figure: (A) SDT3G Glioma cells featuring formation of intracellular vesicles after 48 hours of treatment with 7.5 µg/ml CBD isolate (i), SDT3G Glioma cells featuring absence of intracellular vesicles after 48 hours of concurrent treatment with 7.5 µg/ml CBD isolate and GSK '872 (1 µM) (ii). **(B)** Cytotoxicity of U87MG and J3TBG glioma cells treated with CBD isolate (0 - 20 µg/mL) and VX-765 (50 µM) concurrently for 96 hours. Representative of n = 2 independent experiments expressed as mean ± SD **(C)** Relative growth of glioma cells (Control = 100%) treated with CBD isolate (0 - 20 µg/mL) for 96 hours, sub-groups had the CBD-supplemented media replaced with fresh CBD-free media at 24 and 48 hours. Representative of n = 1 independent experiments **(D)** Glioma cells were measured for baseline extracellular acidification rate for 30 minutes, then treated with CBD at lethal (10 µg/mL) and non-lethal (2.5 µg/mL) concentrations. ECAR was monitored for 500 minutes using the SeaHorse XF24. Representative of n = 2 independent experiments expressed as mean ± SD
